# From Technical Lignins to Sustainable Cement Superplasticizers: A Concise Review

**DOI:** 10.3390/molecules31152558

**Published:** 2026-07-23

**Authors:** Paola D’Arrigo, Luca Leuzzi, Luca Carlomaria Pariani, Letizia Anna Maria Rossato, Luca Schiavi, Stefano Serra, Alberto Strini

**Affiliations:** 1 Department of Chemistry, Materials and Chemical Engineering “Giulio Natta”, Politecnico di Milano, via Bassini 6, 20133 Milano, Italy; luca.leuzzi@polimi.it (L.L.); lucacarlomaria.pariani@polimi.it (L.C.P.); letiziaanna.rossato@polimi.it (L.A.M.R.); 2 Istituto di Scienze e Tecnologie Chimiche “Giulio Natta”, Consiglio Nazionale delle Ricerche (SCITEC-CNR), via Bassini 6, 20133 Milano, Italy; stefano.serra@cnr.it; 3 Istituto per le Tecnologie della Costruzione, Consiglio Nazionale delle Ricerche (ITC-CNR), via Lombardia 49, 20098 San Giuliano Milanese, Italy; luca.schiavi@itc.cnr.it

**Keywords:** sustainable concrete, cement admixtures, high-performance concrete water reducers, bio-based additives, waste biomass valorization, lignosulfonate, circular economy

## Abstract

The construction sector is under enormous pressure to reduce the overall environmental impact of its activities, which, for cement production alone, cause over 2 Gt/yr of CO_2_ emissions (2022). Given the massive scale of concrete production, even minor components such as plasticizers, typically required in quantities of only a few percent of the dry cement mass, correspond to production volumes of many tens of Mt/yr, making their sustainability highly relevant. Lignin is the second most abundant renewable biopolymer on Earth and is currently used in concrete formulations mainly as a low-performance cement plasticizer. Despite this limited application, lignin represents an underexploited but highly attractive raw material for the development of environment-friendly concrete additives, owing to its abundance and availability from industrial biomass residues. The purpose of this review is to provide a specific and up-to-date overview of the various approaches for developing sustainable, high-performance concrete plasticizers based on lignins derived from industrial waste streams. The state of the art in the field is put into perspective by considering the various industrial sources of original technical lignins, the processes that led to their formation, and potential strategies for improving their characteristics. A quick overview of the patent history of the field complements the analysis of the scientific literature. An integral part of this work is a concise introduction to the fundamentals of cement chemistry and rheology, which are essential for understanding the technological requirements for effective concrete admixtures and for enabling cross-disciplinary research between the lignin and cement communities.

## 1. Introduction

Concrete is one of the most globally used man-made materials and is essential in the modern architectural industry for the development of structures, including buildings, bridges, roads, and tunnels. Concrete is a mixture of cement, water, aggregates, and chemical admixtures that can be poured into moulds, allowing for enormous flexibility in structural design. The key component is cement, which acts as a binder, developing a setting reaction within hours that consolidates the structure under construction. World cement production is estimated to be more than 4 Gt in 2020 [1], with a forecast production level for 2030 of more than 8 Gt [2].

The cement industry, however, plays an important role in the problem of climate change caused by greenhouse gases [3]. In 2022 alone, worldwide cement production caused the emission of 2.4 Gt of CO_2_ [4], of which about 50–60% was caused by limestone decarbonation and 30–40% was due to direct combustion of fossil fuels in the plant and for the production of the needed electric power [5]. Part of the CO_2_ emitted is then recaptured by the structures themselves thanks to the progressive carbonation reaction of cement. However, with a recapture flow quantifiable at 0.7 Gt/yr (as an average over the period 2014–2023) [6] and with an estimated value of 0.96 Gt/yr in 2021 [7], this process can only partially mitigate the overall impact of cement production on the atmospheric carbon budget. Current cement and concrete technology remains, therefore, a major source of greenhouse gas emissions globally, contributing over 25% of all current industrial emissions [5].

The entire concrete production chain and the construction sector as a whole are therefore under enormous pressure to reduce the global environmental impact of their activities. In particular for cement production, different mitigation approaches are being studied, including carbon capture and storage, the research of additional cementitious materials for partial replacement of cement, and the development of alkali-activated binders [1]. However, given the scale of the problem, it is clear that a global strategy will be needed to make this industry sustainable in the second half of this century [5,8].

In this context, the relative impact of the production of chemical additives necessary for the production of quality concrete is apparently a minor issue, but in absolute terms, the scale of production is such that even considering only the plasticizers, required in quantities equal to a few percent of the dry cement mass, estimated production volumes of many tens of Mt/yr are obtained. In order to achieve full environmental compatibility in the production of cement and concrete, it is therefore not possible to ignore the development of sustainable methods also for the production of related additives.

Lignin is the second most abundant renewable biopolymer on Earth. It is found in plant cell walls and is widely available as a by-product of the paper and cellulose industry [9]. Lignin is the only aromatic natural polymer and therefore represents a potentially highly sustainable alternative to many synthetic products obtained from the oil industry [10,11]. Potential applications of great interest have been reported and deeply reviewed in the fields of polymers [12,13], functional materials [14,15,16], composites [17,18], coatings [19,20,21], and nanoparticles [22,23,24,25].

Consequently, recent years have seen renewed interest in the use of lignin also in the field of concrete admixtures, which has long been used in the construction industry as a low-performance plasticizer for concrete. Although it is still used in this way in developing countries, in industrialized countries it has substantially given way to high-performance synthetic plasticizers. However, its complex structure, rich in functional groups, allows for extensive modifications and derivatizations [26], making it potentially very interesting for the development of high-performance plasticizers competitive with synthetic products but with a substantially better sustainability profile.

A simple search with a literature database in the 1970–2024 timeframe clearly shows this trend in the academic research activity. Two queries, each composed of three sub-queries associated with the AND operator, were used to perform two separate searches using Scopus (Elsevier) to roughly extract documents pertaining to studies about lignin as a water reducer in concrete. The two searches used the same query structure, the only difference being the search scope of Sub-query 2 (cement OR concrete), restricted to only the title field for Query 1 and extended to abstract and keywords for Query 2 in order to expand the search boundaries (Table 1).

**Figure 1:**
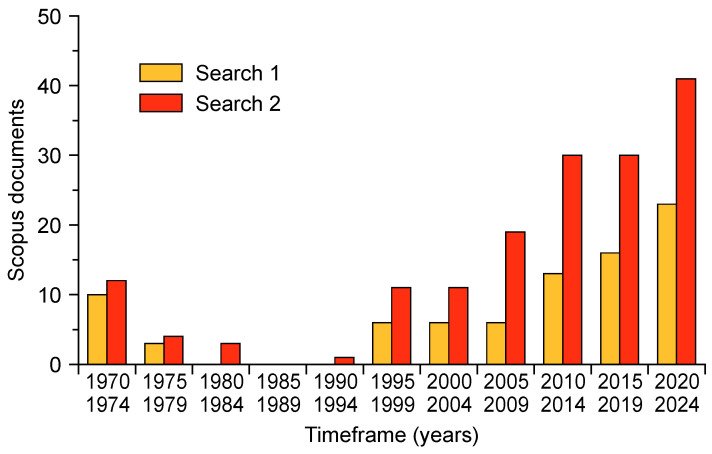
Documents found in Scopus for Search 1 and Search 2 in the overall period 1970–2024, divided into 5-year intervals.

The result of the two searches (Figure 1) demonstrates the same trend, with declining interest in the 1970s, becoming negligible in the 1980s and the first half of the 1990s, and then recovering and steadily increasing from the second half of the 1990s onwards. The increment demonstrated in the new millennium reflects the general increase in awareness of the importance of sustainability and environmental protection issues in all sectors of the modern economy.

The considerable interest in this field is also evidenced by several dedicated reviews published in the literature in recent years, including the work of Breilly et al. [27], specifically aimed at the use of lignosulfonates as superplasticizers in concrete, and the work of Jędrzejczak et al. [28], more generally focused on lignin-based materials and products for sustainable construction. More recently, Lai et al. [29] propose a review of admixtures for environmentally friendly concrete. Moreover, Carvalho et al. [30] propose an original scientometric analysis of the specific field of lignin-based admixtures for cement-based composites.

The development of viable and sustainable high-performance cement plasticizers based on recycled lignin involves the upstream processes for producing technical lignins, the downstream separation and refining processes, and, of course, the techniques and strategies for modifying and derivatizing the lignins themselves. It is important to note that, in this context, even the mere achievement of performance equivalence with synthetic fluidifiers with comparable production costs is to be considered a significant success given the significant advantages obtainable from the point of view of environmental sustainability. This objective fits naturally into the modern concept of a circular economy, where production waste should be reused as a raw material for other processes, thus substantially reducing environmental pressure in terms of resource and energy consumption.

The aim of this review is to provide a concise and up-to-date perspective on the state of research in advanced cement plasticizers based on recycled lignins. Given the intrinsically interdisciplinary nature of this field of research, which combines cement chemistry and technology with the separation and functionalization of recycled biopolymers using advanced bioorganic chemistry methods, specific attention has been paid to illustrating its premises and general context. The first part of this paper is therefore dedicated to describing the basics of cement chemistry and rheology, as well as concrete engineering, which are usually only available in specialized textbooks but are essential for understanding the principles of concrete plasticizer action. The second part delves into the natural origins of lignin and the industrial processes that lead to the generation of recycled lignins as byproducts. These processes, together with the techniques for separating and refining the resulting lignins, form the basis for understanding the raw material on which this field of study is based. The central part of the review analyzes recent studies on possible functionalization routes for technical lignins to transform them into high-performance fluidizers capable of competing with modern synthetic products. To complement the academic research state of the art, a brief overview of the patent history of the sector is also included.

## 2. Cement and Concrete

There are numerous types of hydraulic binders (cements), of which Portland cement is by far the most important and commonly used. Portland cement is produced from a mixture of limestone, silicates, and to a lesser extent, alumina and iron oxides, which are treated at high temperatures in a rotary oven (kiln) to form clinker, which is then finely ground in the presence of a small fraction (a few percent) of calcium sulphate dihydrate (gypsum). Specific grinding aids (e.g., triethanolamine) can be used to optimize this critical step.

Mixed with water, a variable quantity of sand, and coarser inert aggregates, cement powder forms a compound with a fluidity that allows it to be worked directly (e.g., mortars) or shaped by pouring into special moulds (e.g., concretes). Upon contact with water, cement powder gives rise to a series of rather convoluted reactions that lead to the formation of chemical bonds that cause a relatively rapid hardening in the space of a few hours (setting) and then a slower development of high mechanical strength.

### 2.1. Chemistry of Cement

The chemistry of cement hydration is a broad topic that is still the subject of active research today [31]. Cement hydration is a very complex process that depends not only on the chemical composition but also on the physical form (degree of grinding) and the environmental conditions in which it occurs. Furthermore, besides Portland cement, several other types of cements are widely used, including pozzolanic cements (based on Portland cement with the addition of pozzolanic materials, e.g., silica fume, fly ash, and blast furnace slag in various proportions), calcium aluminate cements, and alkali-activated materials (e.g., geopolymers), which constitute an entire class of their own. Even a quick description of the chemistry of the main different types of cement and the combined effects of their interaction with the various additives is largely beyond the scope of the present work. In this section, therefore, only an essential description of the basic processes related to the Portland cement hydration will be given; for further information, it is advisable to refer to the specific technical literature [32,33].

Hydration is characterized by several successive phases that, starting from the first contact with water, generate the setting (within a few hours) and then the final hardening (which lasts several months). The entire process is essentially caused by the dissolution and subsequent reprecipitation of the different chemical species that make up the cement powder, finally resulting in a network of chemical bonds that unite the individual particles to form a single compact mass with high mechanical resistance.

Portland clinker, the main component of Portland cement, is a mixture of several chemical species, the most important of which are tricalcium silicate (Alite or C_3_S in industry jargon, see Table 2), followed by dicalcium silicate (C_2_S), tricalcium aluminate (C_3_A), and dicalcium aluminate (C_2_A).

Contact with water causes the hydration of C_3_S and, to a lesser extent, of C_2_S and free lime, which quickly releases Ca^2+^ and other ions into the aqueous medium. A layer of calcium silicate hydrate (C-S-H) precipitates in a short time on the surface of the cement particles, forming a barrier that slows further dissolution and causes a local increase in the concentration of ions at the surface of the solid particles. C_3_A also dissolves and reacts with Ca^2+^ and SO_4_^2−^ to give Ettringite (AFt), which also precipitates on the cement particles. The precipitation of Ettringite is of particular importance because it inhibits the rapid hydration process of C_3_A with consequent formation of hydrated calcium aluminate precipitates (C_4_AH_19_ and C_2_AH_8_, collectively indicated as AFm), which in turn can cause the rapid setting of the cement (flash setting), preventing its regular workability. Calcium sulphate dihydrate is added to clinker in the preparation of cement specifically for controlling this undesired side reaction.

Furthermore, from the very first moments, the hydration of the surface of the cement particles causes the absorption and dissociation of water molecules on the exposed surfaces of the different minerals that compose them. This leads to the formation of protonation and deprotonation sites and therefore to the formation of surface charges that interact with the ions present in solution deriving from the first phases of dissolution of the cement powder, in particular calcium cations and sulphate anions [34]. The resulting opposing zeta potentials and van der Waals forces between the different suspended cement particles cause attractive interactions that lead to the formation of a percolating network of particles. This evolves into extensive aggregates that sequester part of the mixing water (flocculation).

This first fast phase (pre-induction) takes a few minutes and is followed by a dormant phase (induction) lasting a few hours in which the dissolution and reprecipitation processes are significantly slowed down. At the end of the induction phase, the acceleration phase begins (3–12 h after mixing), where the dissolution reactions resume under the control of the nucleation and growth of the hydration products. The exact nature of the induction process and subsequent recovery is not yet clearly known, with several theories including, among others, the formation of an impermeable hydrate layer (C-S-H), the formation of a Coulomb barrier due to Ca^2+^ adsorption onto the SiO_2_-rich grain surface, and the formation of a supersaturated concentration of calcium hydroxide that inhibits further dissolution of C_3_S [32].

During the acceleration phase, the quantity of hydrates formed and then precipitated (mainly C-S-H and AFt) increases significantly at the expense of the liquid phase, forming a network of chemical bonds that develop at the interface of the cement particles. This network extends over time, causing a progressive loss of the ability to flow of the cement paste until a solid mass is obtained (cement setting phase). Subsequently, the hydration and precipitation reactions gradually slow down (post-acceleration period) until the reactive products are consumed (although a part of the larger cement granules may remain unreacted indefinitely). The matrix of mature Portland cement consists mainly of the C-S-H phase followed by crystalline calcium hydroxide (Portlandite). Minor components include AFt and AFm, which may be present in variable quantities depending on the amount of tricalcium aluminate and iron present in the original material.

A fundamental characteristic of the solidified mass of cement is its open porous structure, the degree of porosity of which depends mainly on the water/cement ratio of the mix and the degree of maturation (as the hydration process proceeds, the porosity decreases). Porosity is a primary factor in determining the final strength of the prepared concrete, with higher strengths obtained from lower porosities. Greater porosity also causes greater permeability to extraneous substances that can cause premature aging of structures (for example, allowing the permeation of chlorides in marine environments, which cause premature corrosion of the metal bars of reinforced concrete). The control of the final porosity of cement matrix is therefore of the greatest importance in concrete technology [32].

### 2.2. Rheology of Cement Paste and Concrete

The rheology of a fresh cement paste or concrete evolves in time from a paste-like consistency, which allows it to be modelled in the desired shape (typically with casting systems), to a rigid, rock-like structure. The rheology evolution process can be subdivided into several subsequent phases that start with the first contact of water with the cement powder. At time zero, the system is essentially constituted by a suspension of solid particles in water, and the rheology is mainly determined by the water/cement ratio and by the particle size distribution and geometry. After a few minutes, the flocculation process causes the formation of cement particle aggregates that increase the paste viscosity. At this stage the process is mainly reversible because the particle aggregates can be disrupted by the application of mechanical energy (e.g., ultrasound or intensive mixing). During this early phase, the cement (or concrete) mix is thus characterized by a good plasticity (also indicated as “workability”) in which the paste can be mixed, poured, and cast. The cement hydration, however, causes a progressive precipitation of hydrates on the particle surface, progressively increasing their dimensions and reducing the water/solid ratio. Moreover, inter-particle chemical bonding starts to form between the deposited layer of hydrated products. Differently from the previous case, inter-particle chemical bonds cannot be broken by mixing, and thus this causes an irreversible increase of viscosity. The progressive build-up of inter-particle bonds (the stiffening phase), a process that accelerates at the end of the dormant phase of the hydration process, causes a continuous increase of the particle aggregate size that eventually results in the formation of a percolating bond network that encompasses the whole mass (the setting phase). At this point, the system loses its ability to flow, and a solid mass with stable form is established. The mechanical strength at this stage is still low because the particles are bonded only in small contact zones and a relatively small force can break the network, causing the collapse of the formed structure. However, during the successive hardening phase, the hydration process continues, and the precipitation of further hydration products increases the inter-particle contact surface and allows the development of the full mechanical strength of the solid mass [33].

The mechanical characterization of the evolving cement paste is strictly dependent on the specific process phase. During early hydration, the flow properties of the paste or concrete can be described in terms of the relation between the shear rate and the shear stress. Particularly interesting are the plastic viscosity (i.e., the ratio of the shear stress increase with the increase of the shear rate) and the yield stress (i.e., the limit of the shear stress for the shear rate approaching zero). Moreover, it can be very interesting to characterize the thixotropic properties of the mix (i.e., its ability to recover the former structure following a disruption caused by applied shear) and its viscoelastic properties (i.e., splitting the viscous and elastic components). These can be measured with interval rheometry (for thixotropy measurements) or with oscillatory rheometry (for both).

Typical simpler technical tests are based on the measurement of the deformation of a sample cast in a semicone mould by application of mechanical stresses or simply by self-flow caused by weight. Examples are the flow table (typically applied to mortars) for the former and the Hägermann flow (for cement pastes) and slump test (for concretes) for the latter. Other methods based on the flow velocity or spread of the paste through a well-defined geometry are also described.

The determination of the setting time is somewhat arbitrary because it is a continuous process and its value is strictly dependent on the measurement method. A common procedure is the Vicat test, carried out by measuring the penetration of a 1 mm^2^ needle loaded with 300 g in the cement paste at fixed time intervals. The beginning and end of the setting are typically defined by the time the needle penetrates to a certain depth in the cementitious paste.

The solidified mass is characterized mainly by its mechanical strength (typically as maximum compressive or flexural load before structure collapse) and by its shear modulus (the ratio of shear stress and shear strain). These properties evolve during the hardening phase (a process that lasts for months) and are typically measured at 2, 7, and 28 days from casting [32].

### 2.3. Water Reducing Agents

In construction practice, the control of the characteristics of fresh concrete is of supreme importance to optimize both the deployment process and the final characteristics of the built structures. A wide range of admixtures were developed to modulate the viscosity, the setting time, the air entraining, the shrinkage, and other physical and chemical parameters of the mix in order to fulfil all the requirements of modern, high-performance projects [32].

Among others, workability (which deeply impacts the casting and the utilizable delivery techniques) and final mechanical resistance (that determines the characteristics and the project of the manufact) are two of the most important parameters that define a concrete mix. Both are strongly influenced by the water-to-cement (*w*/*c*) ratio but in opposite directions, with more water allowing enhanced workability (decreasing the mix viscosity) but reducing the mechanical performance of the final product (increasing the porosity of the hardened binder). Higher *w*/*c* ratios moreover typically increase the shrinkage during the hardening process and enhance the concrete permeability, facilitating the migration of detrimental chemical species such as carbon dioxide and chloride ions.

Water reducing agents are thus of particular importance because they allow reduction of the *w*/*c* ratio, maintaining the same workability or, alternatively, enhance the workability maintaining the same *w*/*c* ratio. A balance of the two effects can be achieved by proper design of the concrete mix. Water reducers can be roughly divided into normal water reducers (ordinary plasticizers, allowing up to 10–15% of water reduction) and high-range water reducers (superplasticizers, with up to 30% and more water reduction capability).

The mechanism of action of ordinary plasticizers is mainly based on a complex interaction between the surface charges on the cement particles formed following the hydration process, the charges present on the plasticizer chain, and the ions in solution, among which Ca^2+^ is particularly important. The generally accepted basic mechanism involves the adsorption of the plasticizer’s polymer chains, typically comprising negatively charged functional groups such as carboxylic and sulfonic acids (ionized in the alkaline environment caused by the cement matrix), onto the surface of the cement particles, which are characterized by the presence of numerous positively charged sites. Following adsorption onto the cement particles, the plasticizer chains form a stable electrostatic barrier, generating repulsive forces that tend to separate the particles, inhibiting direct mechanical interactions and flocculation, and thus improving the flow characteristics of the mix [32].

**Figure 2:**
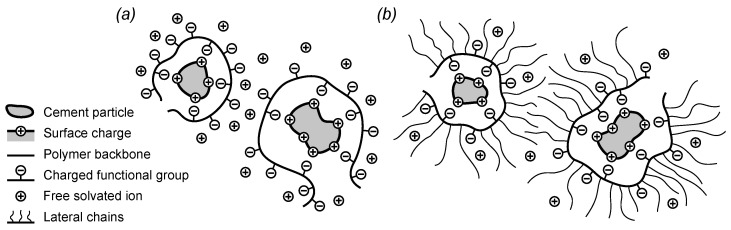
Mechanism of action of typical cement plasticizers (simplified). Ordinary plasticizer, based mainly on electrostatic repulsion (**a**), and high-performance superplasticizer (PCE) based mainly on the steric hindrance of the side chains (**b**).

Synthetic superplasticizers are polymer systems specifically designed for the fluidization of cementitious products. Typical examples are sulfonated melamine-formaldehyde condensates, sulfonated naphthalene-formaldehyde condensates, and polycarboxylate ethers (PCEs). The latter are high-end superplasticizers with a structure that includes a main chain (backbone) rich in carboxylic acid groups and several nonionic side chains. While the main chain is adsorbed onto the surface of the cement particles through direct Coulomb interactions, the side chains are not adsorbed and interact with the aqueous medium, providing significant steric hindrance that keeps the particles separated (Figure 2). The characteristics of these plasticizers therefore are highly dependent both on the number of carboxylic acid groups that promote adsorption and on the structure of the side chains (length, branching, and composition), which provide steric stabilization of the suspension [35].

## 3. Lignin

Lignin is the second most abundant plant resource after cellulose and represents the major aromatic biopolymer constituting 15 to 33% of the cell wall of terrestrial plants [36,37]. Natural lignin is present in lignocellulose backbones and doesn’t exist isolated [38]. Its structure is highly branched and irregular, with a complex three-dimensional structure composed of phenylpropanoid units. Instead, technical lignins are mainly obtained as a waste product in the production of paper and biofuels, where they are separated from cellulose and hemicellulose in various high-volume processes [39,40]. Moreover, all the residues from agriculture or the agri-food industry (such as sugarcane bagasse [41], corn cobs [42], rice husks [43] and brewer’s spent grain [44]) contain lignin, which constitutes around 5–25% of the total biomass. Worldwide, about 100 Mt/yr of technical lignins are generated as industrial by-products [45,46] and mainly disposed of or burned for heat production, with only a minor part recovered for further processing. The expansion of lignin industrial valorization is therefore a highly attractive target from a circular economy perspective.

### 3.1. Natural Lignin

**Figure 3:**
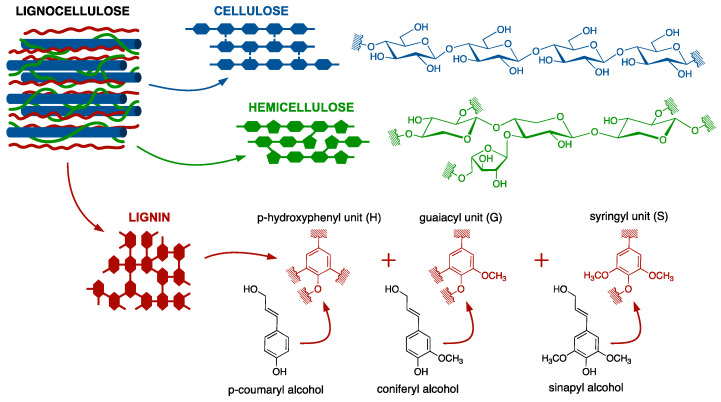
Lignocellulose structure and lignin monolignols.

The essential function of lignin is to provide a structural skeleton with mechanical strength, hydrophobicity, and resistance to microbial degradation in plants. In nature, the complex polyphenolic structure of lignin is strictly bound to cellulose and hemicellulose in the lignocellulosic biomass (see Figure 3).

All three polymers interact with each other, and this confers the main characteristics to a plant: flexibility, strength, stiffness to the cell walls, resistance against insects and pathogens, and recalcitrance to enzymatic degradation [47]. Lignin structure is built with a highly complex methoxylated phenylpropane skeleton mainly obtained by the oxidative coupling of phenylpropane units called monolignols: *p*-coumaryl alcohol, coniferyl alcohol, and sinapyl alcohol, containing zero, one, and two methoxy groups, respectively. The corresponding units in lignin, reported also in Figure 3, are known as *p*-hydroxyphenyl (H), guaiacyl (G), and syringyl (S) units [48]. Native lignins possess an intrinsic heterogeneity due to the botanical origin of the biomass that distinguishes the different types of lignins. The lignin monomer ratios and the bonds formed during the polymerization process depend on the plant species and growth conditions, giving significant variability in the skeleton structure and the physicochemical properties depending on its natural origin, as reported in Table 3 [49].

The intrinsic H/G/S ratio severely influences the cross-linking density and functional group availability of the isolated lignin, and for sustainable cement plasticizer development, these native differences are crucial. For example, in G units, the absence of methoxyl groups at the C5 position renders softwood lignins highly prone to condensation reactions during the extraction, and that results in a more rigid, highly branched three-dimensional network. Instead, in hardwood lignins, which are S-enriched, the C5-condensation is suppressed, and that condition allows a more linear polymer with a higher density of aliphatic hydroxyl groups.

### 3.2. Technical Lignins

**Figure 4:**
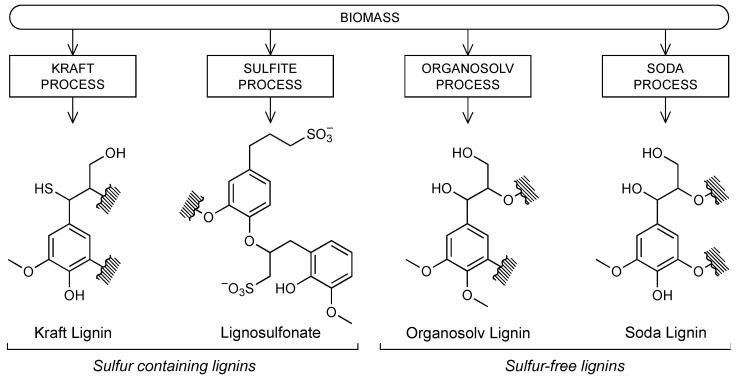
Main biomass fractionation processes and a representative skeleton element of the corresponding recovered technical lignins.

Technical lignins are extracted from natural biomass as by-products of various industrial processes (typically polysaccharides-oriented) and are therefore modified to varying degrees from their natural form [50,51]. Technical lignins present different properties according to the extractive method used, which could be divided into two main groups: sulfur-containing or sulfur-free lignins [52]. Sulfur-containing lignins are mostly obtained from the paper and pulp industry, and Kraft, sulfite, and hydrolyzed lignins are the most common ones. Sulfur-free lignin is a more recent emerging class of lignins that are obtained with solvent pulping or soda pulping processes. The different processes are summarized in Figure 4.

#### 3.2.1. Kraft Lignin

Globally, the wood Kraft pulping constitutes the most widely used process for the production of paper [53]. Kraft lignin is obtained by the dissolution of the lignocellulosic biomass using sodium hydroxide and sodium sulfide, which are responsible for bond cleavage between lignin and cellulose. This process converts the woody and non-woody materials into pulp and a waste by-product (black liquor) that is then mixed with an acidic solution to precipitate and recover lignin. This process accounts for about 85% of the global technical lignin production. Kraft process is remarkably effective in lignin removal and, as a result, lignin is degraded into water-insoluble compounds which are less versatile than lignosulfonates. Moreover, Kraft lignin is a sulfurous lignin because of the use of NaOH and Na_2_S in the delignification, but the final sulfonation degree is not high; in fact, only 1.5–3% (*w*/*w*) of the final backbone is sulfonated [54]. This lignin is largely destined for combustion for energy production for the necessities of their own industrial production site, with a minor part destined to further processing for valorization.

#### 3.2.2. Lignosulfonate

The second important industrial process for the production of lignin is constituted by the Sulfite process, which, by means of sulfur dioxide combined with ions of calcium, sodium, magnesium, and ammonium, leads to the sulfonation of lignin, producing a lignosulfonate whose essential property is water solubility. Furthermore, sulfite lignin has higher sulfur content (3.5–8%), and for those reasons it represents the main biopolymer used as concrete dispersant [55]. Lignosulfonates are widely used as ordinary water-reducing agents because they are widely available and constitute a low-cost, renewable, and sustainable resource. Unmodified lignosulfonates have, however, moderate water reduction capabilities (falling in the class of ordinary water reducers).

#### 3.2.3. Organosolv Lignin

Organosolv lignin is sulfur-free and is obtained thanks to a pulping process based on organic solvents, like methanol or ethanol [56,57]. The most common extraction process utilizes ethanol/water, acetic acid, or formic acid. One of the most important advantages is that this method allows the extraction of cellulose and hemicellulose, which in turn can be used in a different downstream process, improving biomass valorization. Despite these benefits, until now, organosolv lignin has not been widely adopted in large-scale production [58].

#### 3.2.4. Soda Lignin

Soda lignin is obtained thanks to soda pulping processes conducted especially on agricultural wastes. The lignin content is low, so only 10–15% of soda is required, and the addition of catalytic quantities of anthraquinone can stabilize the carbohydrate fraction [59]. It can become important and largely used in the second-generation biorefineries because of the loss of sulfur, but also because of the lower delignification rates. The Soda lignin molecular weight is typically lower than that of Kraft and sulfite lignins [56,60].

### 3.3. Advanced Lignin Fractionation Processes

Technical lignins derived from current industrial processes are typically characterized by low homogeneity, with characteristics strictly dependent on the original plant species, the specific growth conditions of the processed biomass, and obviously the type and operating conditions of the fractionation process itself.

There is therefore considerable interest in developing advanced fractionation processes (both of the original biomass/waste stream and of already isolated technical lignins) that provide improved results in terms of the chemical and physical characteristics of the final lignin, with particular reference to molecular mass dispersion [61,62,63]. The most common technologies to separate lignin molecules into fractions with low polydispersity and well-defined properties could be classified in the following groups: precipitation by pH modifications, fractionation by solvents, separation by ultrafiltration, applications of resins, use of ionic liquids (ILs), and deep eutectic solvents (DESs).

Precipitation by pH is one of the more deeply investigated systems for black liquor fractionation [64,65]. Moreover, the precipitation by pH as well as the extraction performed by solvents exploits and fractionates the raw material depending on its chemical composition. Lignin presents a very broad and complex composition in terms of functional groups, a characteristic that allows the obtainment of different fractions by performing a pH gradient precipitation, as this method exploits the different protonation abilities of various functional groups, resulting in a selective precipitation of the material versus pH values.

Solvent fractionation, on the other hand, exploits the specific solubilization properties of different lignin fractions, either by performing a series of independent extractions (for example, on isolated technical lignins [66]) or by performing a series of controlled precipitations with the addition of antisolvents (for example, on spent liquor wastes [61]). The use of organic solvents can impact the environmental and energy sustainability of the process, and it is therefore necessary to consider the energy and operational aspects of recovering and recycling the solvents used.

Ultrafiltration fractionation allows for the direct selection of different fractions based primarily on molecular weight and can be applied to technical lignin solutions [67] or directly to industrial waste streams [68]. This fractionation process is undoubtedly highly attractive from an environmental perspective and can also be applied in a series of cascading steps to achieve simultaneous differentiated fractionations. Its main critical point lies in the tendency of filter membranes to foul, which must be controlled with appropriate measures such as tangential-flow filtration [69].

Fractionation of solubilized lignins by sorbents is based instead on the dissimilar tendency of the various fractions to be adsorbed by a solid medium [70,71]. Sorbent fractionation is particularly interesting for the separation of small phenolic compounds in green processes, allowing the operation at low temperatures and with relatively limited volumes of solvents.

On the other hand, ionic liquids (ILs) and deep eutectic solvents (DESs) have been studied in recent years for biomass fractionation and lignin isolation [72,73,74]. These methods are highly attractive from an environmental perspective and also offer a wide range of options for tuning operating conditions to optimize the separation process. Of particular interest in this regard is the possibility of having a significant number of different DES formulations thanks to the large number of binary or ternary combinations obtainable from even a limited set of components. Moreover, a combined microwave/DES-mediated fractionation approach has been recently explored as a mild, tunable, and sustainable alternative to conventional methods [75,76].

In recent years, interest has grown in the search for combined solutions capable of exploiting the strengths of different fractionation techniques by combining them in a single integrated process, such as the ionosolv-organosolv process [77], organosolv-ultrafiltration process [78], and DES-IL process [79,80] (for biomass fractionation) or solvent extraction-ultrafiltration process [81] (for technical lignin fractionation).

The development of advanced lignin fractionation methods will undoubtedly be a very active research topic in the coming years, both in terms of developing the actual separation methods and in scaling up from the laboratory to the plant, where it is necessary to take into account many aspects that are negligible at small scale [82].

## 4. Lignin as Cement Dispersant

Lignosulfonates recovered from industrial processes were one of the first water-reducing agents applied in concrete technology, having been in use since the 1930s. Thanks to their low cost and wide availability as a by-product of the pulping industry (spent liquor), they are still used today, particularly in emerging countries, despite their inferior performance compared to modern synthetic products. A substantial part of the solid fraction of spent liquors consists, however, of sugars, which have a strong retarding effect on cement setting. This is usually an undesirable effect that can be mitigated by purification processes to reduce the total carbohydrate content. The performance of lignosulfonates is also strongly dependent on molecular weight, with better performance achievable from fractions with higher molecular weight. Commercially available lignosulfonates have a certain capacity to trap air in the concrete being processed, which can become significant at high dosages. However, this can be effectively counteracted by adding small doses (a few percent by weight relative to the lignosulfonate) of defoaming agents, such as dibutyl phthalate or tributyl stearate. Thanks to the availability of commercial purification and fractionation methods, lignosulfonate-based products with a defined range of molecular weights and residual sugar content are now commonly found on the market, thus enabling reproducible performance. Commercial lignosulfonates are available as sodium, calcium, or magnesium salts. Sodium lignosulfonate is generally the preferred form for best performance and solubility, while calcium salt is more economical [32,35,83]. Lignosulfonates used as dispersants in the preparation of concrete demonstrate a relatively limited capacity for water reduction, usually 8–10% with typical dosages of 0.1–0.3% *w*/*w* relative to cement [35].

Lignosulfonates act as ordinary cement plasticizers (Section 2.3), where the negative charges of the sulfonic groups (ionized in the alkaline environment of the cementitious matrix) promote electrostatic interaction with the cement particles and at the same time cause the formation of an electrostatic barrier that inhibits direct particle–particle interactions.

### 4.1. Lignin Chemical Modifications

**Figure 5:**
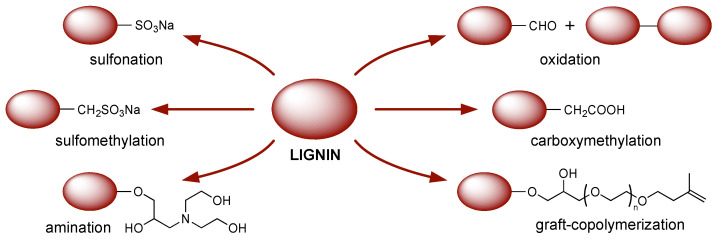
Main lignin chemical modifications.

In the past, and more intensively in recent times, numerous studies have been carried out to improve lignin performance, in particular by modifying its molecular structure. In fact, unmodified technical lignins could generally offer a limited dispersing capacity because of their low charge density and heterogeneous structure. One of the two main strategies explored especially in the past involves the insertion or formation of additional negatively ionizable functional groups (e.g., sulfonic or carboxylic acid groups) to increase both solubility and plasticizing activity. The second strategy, which has seen numerous studies in the new millennium, involves the insertion of large polymer side chains with the primary purpose of inhibiting particle–particle interactions through steric repulsive forces. The essential chemical modifications discussed below are summarized in Figure 5.

#### 4.1.1. Introduction of Sulfonated Groups

The introduction of sulfonated functional groups into the lignin skeleton can be provided by two main chemical modifications: sulfonation (performed in the sulfite process, Section 3.2.2) and sulfomethylation.

The introduction of sulfonic groups by sulfomethylation into unsulfonated lignins or into lignins with an insufficient degree of sulfonation (for example, those derived from the widely implemented Kraft process) makes them suitable for application as cement plasticizers [84]. The sulfomethylation is generally performed using a mixture of sulfite and formaldehyde and leads to the introduction of a sulfonated methyl group (–CH_2_SO_3_) into the lignin backbone, with a particular reactivity of the ortho positions to phenolic hydroxyl groups, which constitutes the activated aromatic sites. Compared to conventional sulfonation, sulfomethylation allows for a higher and more homogeneous incorporation of sulfonic functionalities, leading to improved water solubility and more consistent performance.

**Figure 6:**
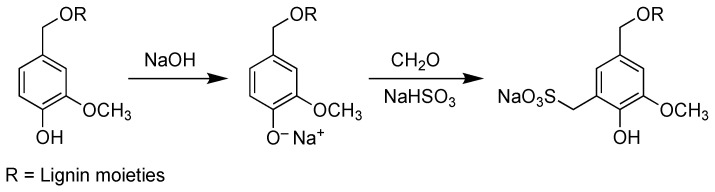
Lignin sulfomethylation using a mixture of sulfite and formaldehyde.

Kamoun et al. proposed the sulfomethylation of a lignin extracted from esparto grass (*Stipa tenacissima*) according to the soda process [85]. The sulfomethylation process was carried out in a conventional way, by treating lignin with a mixture of sulfite and formaldehyde (see Figure 6), and its performance as a cement dispersant was tested on a Tunisian Portland cement.

During the process, the aliphatic groups on lignin side chains were sulfonated because of an excess of sulfite in the reaction, while the lignin aromatic nuclei were sulfomethylated, thanks to the reaction of sulfite with formaldehyde. The degree of sulfonation depended on the reaction conditions (with particular reference to the formaldehyde-to-sulfite molar ratio), and this influenced lignin solubility, molecular weight, and hydrophilic characteristics. Optimal results were obtained when the reaction was carried out under the following conditions: molar ratio of formaldehyde to sulfite of 0–0.8, at a pH of 7–9 for 3–6 h, with a sulfite concentration of 20–50% (*w*/*w* sulfite/lignin). Under these conditions, the obtained lignin presented a relatively high degree of sulfonation, high molar weight, and narrow molar weight distribution. The plasticizing ability of this modified lignin was attributed to its molecular weight and anionic charge density. Its optimal dosage as cement dispersant was 0.4% (*w*/*w* lignin/cement), leading to a good plasticizing effect and a reduction in water content, maintaining the cement workability.

Huang et al. obtained lignosulfonates by sulfomethylation of an alkaline lignin (AL) and an enzymatic hydrolysis residue (EHR) recovered from an alkali-treated bamboo residue, and compared their performance on cement paste with respect to commercial lignosulfonates in terms of cement paste fluidity, water reducing rate, and concrete strength [86]. In general, even at optimum conditions, AL showed a higher sulfonation degree than EHR, because of its higher content in reactive sites (free C5 position of lignin). This could be explained by AL’s lower molecular weight and lower condensation. In fact, the density of sulfonic groups in sulfonate AL and sulfonate EHR was 1.6 mmol/g and 1.0 mmol/g, respectively. Therefore, lignosulfonate AL showed better dispersant activity than commercial lignosulfonates and sulfonate EHR, improving cement paste fluidity and reducing the water content needed. However, sulfonate EHR had a more positive effect on the compressive strength of concrete, probably because of the minor air content trapped in the paste. Finally, these results suggested that the lignosulfonates obtained by the transformation of lignins recovered through biorefinery processes have a potential application in improving concrete properties, meeting the industrial standards required for commercial water reducers.

Recently, Zandonadi Nunes et al. also produced lignosulfonates by sulfomethylation of eucalyptus Kraft lignin and heat-treated Kraft lignin, and tested their activity as concrete dispersant on Brazilian cement (with high content of clinker) in comparison with a commercial admixture of lignosulfonates [53]. Both the produced lignosulfonates presented a high sulfonation degree, which increased their water solubility from 7.2% to 98.8%, compared to Kraft lignin. When both Kraft lignosulfonate and heat-treated lignosulfonate were added to the concrete, they showed an increase in concrete workability, allowing a reduction in water consumption. However, no significant improvements in concrete workability were observed when the heat treatment was performed, permitting its elimination from the biorefinery process, reducing its overall costs and increasing its economic feasibility.

**Figure 7:**
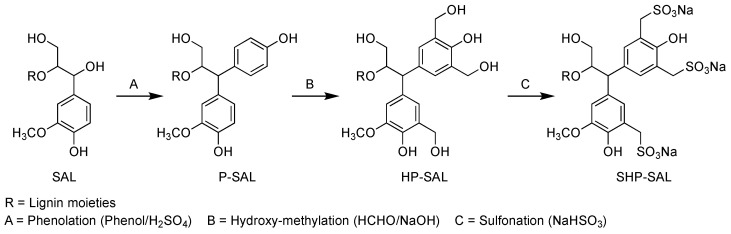
Synthesis of SHP-SAL from acid-precipitated Kraft lignin (SAL).

Syahbirin et al. [87] have reported the modification of Kraft lignin from eucalyptus, isolated from Kraft black liquor with acid precipitation (SAL), using a three-step process (Figure 7). In the first step, the authors introduced a phenolic group into the SAL structure, which was then functionalized by hydroxymethylation. The sulfonic groups were then introduced by treatment with sodium hydrogensulfite to obtain a sulfonated hydroxymethylated phenolized sulphuric acid lignin (SHP-SAL). This allowed them to substantially increase the degree of sulfonation, resulting in a water-soluble product. These modifications were performed in order to improve lignosulfonate dispersibility in the concrete mixture, which was influenced by lignin structure, sulfur content, and molecular weight. Its dispersant activity was tested on gypsum paste by monitoring lignin dispersibility and cement fluidity, and compared to that of commercial sodium or calcium lignosulfonates and sulfonated naphthalene formaldehyde condensate (SNF, a high-performance commercial plasticizer). SHP-SAL showed great dispersant properties, better dispersibility than commercial calcium/sodium lignosulfonates, and equal performance to SNF.

#### 4.1.2. Oxidation

The oxidation reaction is of high interest because it causes, in the lignin backbone, the introduction of carboxylic acid functionalities, as well as additional hydroxyl groups or carbonyl groups coming from the oxidation of alcohol moieties. These processes also lead to the modification of the existing side chains and the aromatic rings. The main issue is to set up the optimization of the oxidation process in order to achieve the right oxidation degree without the occurrence of excessive degradation.

**Figure 8:**
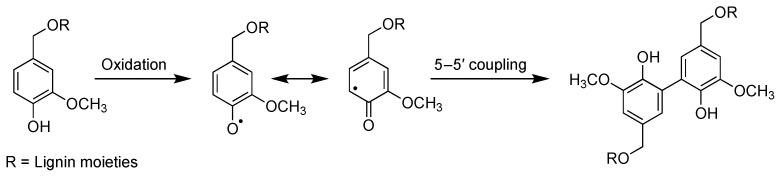
O_2_-mediated oxidation process with 5–5′ radical coupling reaction.

Different oxidation agents can be exploited, such as oxygen, ozone, or hydrogen peroxide. Typically, oxidation is performed to increase lignin water solubility by the addition of acidic groups. For example, Kalliola et al. developed an alkali-O_2_ oxidation process on wheat straw Soda lignin, which allowed the increase of negatively charged groups and lignin condensation [88]. A two-step oxidation was performed to first polymerize and then oxidize lignin, and pH was found to be the most crucial factor to obtain lignin with high molecular weight. In fact, at pH below 12, the hydroperoxide intermediates were protonated and decomposed to their phenoxy radicals, which spontaneously performed a 5–5′ coupling reaction, without consuming the phenolic groups in lignin (Figure 8). This condensation was favoured by high lignin concentrations, since the phenoxy radicals were close to each other. Furthermore, the dissolution and diffusion of oxygen, which was mainly responsible for degradation reactions, was blocked in the lignin solution because of its high viscosity. After this first oxidation, the lignin-free phenolic groups reacted with oxygen, also allowing the increase of the negatively charged moieties. This modified lignin was tested on two types of cement (an ordinary Portland cement and a blended limestone/slag cement), using commercial plasticizers as reference (a lignosulfonate and three PCEs). The modified lignin showed better performance than the commercial lignosulfonate, also without introducing air in concrete and without reducing concrete compression strength.

The same group proposed the oxidation technology also on Kraft lignin, through an innovative method for lignin recovery from black liquor, which avoided the use of sulfuric acid and allowed the production of a high-performance cement dispersant. In fact, in the Kraft process, lignin is usually recovered from the black liquor by CO_2_ addition, which decreases the pH from 13–14 to 9–10, causing lignin phenoxy groups to protonate and lignin precipitation. Then, lignin is recovered by filtration and re-suspended in water by sulfuric acid addition, causing the presence of an excess of sulfur, which must be removed by washing with massive amounts of NaOH. This method has the drawback of presenting some environmental issues, together with high costs, which limit the application of this technology. To overcome these problems, Kalliola et al. proposed the oxidation of lignin after its precipitation with CO_2_ by using oxygen at alkaline conditions, and the oxidized lignin was then recovered through a membrane filtration technology [89]. This method allowed for concentrating the lignin content and recovering and recycling the present chemicals (Na and S) to produce high-performance admixtures, reducing the overall costs and environmental impact. This simplifies lignin recovery, and no excess sulphur is introduced into the mill cycle. As the unwashed lignin is alkaline in nature, the need for fresh alkali in the lignin oxidation is reduced. Moreover, oxidized white liquor, readily available in the mill, can be applied as a partial source of alkali. Finally, membrane filtration of the oxidized lignin solution enables the recirculation of sodium and sulphur back to the chemical cycle and provides a concentrated lignin product. Furthermore, the study demonstrates that the oxidized lignin dispersants produced through this method are competitive with commercial oil-based plasticizers in performance. This integrated process not only streamlines lignin recovery and oxidation but also contributes to more sustainable and cost-effective operations within Kraft pulp mills. The obtained lignin was tested in mortar and concrete, using three commercial plasticizers as reference (lignosulfonate, polycarboxylate ether, and naphthalene sulfonate). The results showed a water reduction of 12% by using only 0.36% lignin (*w*/*w* lignin/cement), providing also an even higher slump than in the case of the reference concrete mix.

Sutradhar et al. produced green cement plasticizer in a one-step aerobic oxidation of Kraft lignin (KL) in the presence of KOH [90]. In this study, optimizing the reaction conditions led to the maximum carboxylic acid group and charge density of 2.6 mmol/g and 1.9 mequiv/g for oxidized lignin, respectively. The NMR, FTIR, and XPS analyses quantified the alterations in the structure of KL after the oxidation reaction. The molecular weight analysis revealed that lignin depolymerization would depend on the temperature, lignin amount, and KOH concentrations. The oxidized lignin, characterized by a higher content of carboxylic acid groups, absorbs on the cement particles more readily than a commercial plasticizer, which increased the flowability of the pastes. The compressive strength of cement was improved much more by using oxidized lignin instead of a commercial plasticizer and lignosulfonate. This study suggested that the one-pot KOH-mediated oxidation of Kraft lignin can be a promising approach to producing a green plasticizer for cement application.

#### 4.1.3. Carboxymethylation

Another widely investigated chemical modification is carboxymethylation, which is characterized by the reaction of lignin hydroxyl moieties with carboxymethyl functionalities [91]. The changes in the hydroxyl and carboxyl group content are intrinsically influenced by several critical parameters, including alkaline medium concentration, carboxymethylation reagent concentration, reaction time, and temperature. This process typically involves the reaction of lignin with monochloroacetic acid or monochloroacetate sodium salt under alkaline conditions, introducing carboxymethyl groups (–CH_2_COO^−^) in the lignin skeleton onto phenolic and aliphatic hydroxyl sites. The incorporation of anionic residues contributes to increasing the water solubility and charge density of lignin, leading to an enhancement of its ability to interact with and adsorb onto cement particle surfaces [92]. As a result, carboxymethylated lignin promotes electrostatic repulsion between cement particles after adsorption of the modified lignin on their surface and improves the workability of cement pastes because it acts as an effective dispersing agent, preventing flocculation, enhancing particle dispersion in aqueous suspensions, and reducing self-aggregation [93]. Stronger interactions during cement hydration with calcium-rich phases can take place, influencing both the rheological behavior of cement pastes and the stability of particle suspensions. Obviously, as already stated, the results of the process of carboxymethylation are highly related to the lignin typology, the amount, and the distribution of the functional groups which could be derivatized.

For example, Akond et al. proposed the introduction of carboxylic acid on the lignin extracted with deep eutectic solvents from secondary agricultural residues of food processing, in particular sugarcane bagasse [9]. Carboxymethylation of this lignin was performed by mixing lignin with solid NaOH and deionized water. Then, monochloroacetic acid solution (37.5% *w*/*w*) was added, and the mixture was allowed to react for 90 min. Vacuum filtration separated the carboxymethylated lignin, which was subsequently washed with ethanol several times and then dried. This lignin was found to be a feasible cement plasticizer candidate, increasing the workability of cement paste when used at 0.3% (*w*/*w* lignin/cement).

Carboxymethylation is an attractive strategy because it relies on relatively simple chemistry, uses widely available reagents, and can be applied to different types of technical lignins, including Kraft and soda lignins. Furthermore, the presence of multiple carboxylate functionalities provides a versatile platform for further chemical tailoring or graft copolymerization. For these reasons, carboxymethylated lignin derivatives are currently considered promising candidates for the development of more sustainable, bio-based plasticizers for cement and concrete applications.

#### 4.1.4. Amination

**Figure 9:**
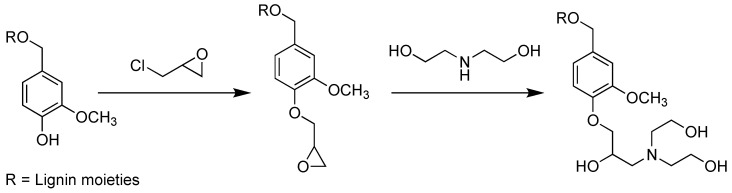
Diethanolamine-modified lignin (DML) synthesis.

Slightly off-topic with respect to the subject of this review, an interesting application as a grinding aid in cement production was studied by Li et al. by derivatizing lignin with diethanolamine [94]. The diethanolamine-modified lignin (DML) was synthesized by lignin, 2(chloromethyl)oxirane, and diethanolamine, and used for the production of Portland cement (Figure 9). The results showed that DML improved the grindability and particle size distribution efficiently, and the cement mortars made with DML exhibited excellent mechanical properties. In contrast to unmodified lignin, it was found incidentally that DML not only had excellent grinding performance but also showed a strong and unique retarding effect. The retarding mechanism is mainly attributed to steric hindrance of molecular structure and electrostatic interactions of anchoring groups adsorbed on the surface of cement during the hydration process, and the synergistic effects retarded further hydration of cement.

#### 4.1.5. Graft-Copolymerization

Graft-copolymerization represents a relatively new method to derivatize lignins for application as plasticizers, which aims to increase lignin molecule steric hindrance and enhance its potential as a water reducer in concrete preparation. Different strategies have been reported in the literature.

**Figure 10:**
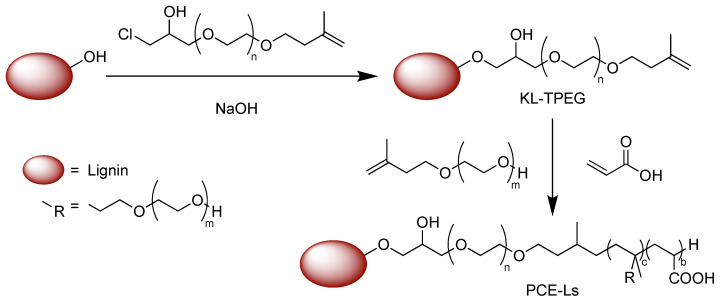
Synthesis of PCE-Ls via KL-TPEG.

Zheng et al. [95] propose a two-step process that starts by grafting a chlorinated derivative of isopentenol polyoxyethylene ether (TPEG) onto Kraft lignin (KL) to form a basic macromonomer (KL-TPEG). KL-TPEG is then used as a building block for the synthesis of a polycarboxylic ether (PCE) dispersant via free-radical polymerization with acrylic acid and TPEG (Figure 10). The final product (PCE-Ls) is a cement dispersant based on the interaction of lignin, polycarboxylic chains, and nonionic side chains. The paper describes the synthesis of three derivatives with different lignin contents (2%, 10%, and 30%) and a reference polycarboxylate (lignin-free) prepared with the same process (PCE-B). The synthesized products demonstrated similar behavior in the dispersion of a cement paste, while 30% of the products showed greater dispersion than PCE-B in the slump test with concrete.

In a subsequent work [96], the same group synthesized a series of Kraft lignin derivatives with TPEG (KL-TPEG) with lignin/TPEG ratios ranging from 1:1 to 1:5. The derivatives with a higher fraction of TPEG demonstrated better dispersing ability in rheological measurements.

**Figure 11:**
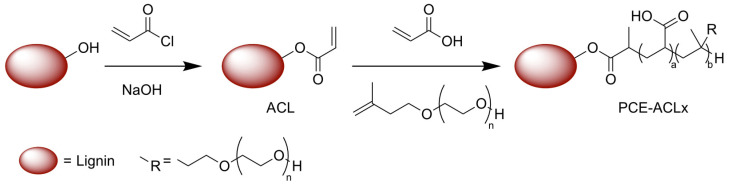
Synthesis of PCE-ACLx.

Zhong et al. suggest in a subsequent work that the PCE-Ls dispersants had, however, elevated hydrophilicity, provoking a reduced effect in decreasing concrete viscosity [97]. To overcome this problem, the authors propose a modified synthesis in which the bridge between lignin and the grafted PCE chain is not made of TPEG but of an acrylate ester. In the proposed process, Kraft lignin was thus esterified with acryloyl chloride (AC) to prepare the lignin-based unsaturated ester (ACL) that was used to replace TPEG macromonomers in the following PCE synthesis. Finally, ACL and TPEG were copolymerized with acrylic acid to prepare the final PCE-based lignin plasticizer (PCE-ACLx, Figure 11).

Different ACL/TPEG mass ratios were tested, and the 1:99 ratio (PCE-ACL1) showed a positive dispersion effect. In particular, at a PCE-ACL1 dosage of 0.2% (*w*/*w*) and *w*/*c* ratio of 0.29, the cement paste fluidity was 283 mm (mini-slump cone method), and at *w*/*c* of 0.35 the emptying time of fresh paste was 13.9 s (Marsh cone method), showing a good viscosity-reducing effect. Compared to common PCE, the produced PCE-ACL had lower adsorption capacity on cement particles, but larger steric hindrance and gas-inducing properties.

Another possible option is grafting lignin with anionic polymers, as Childs et al. did by grafting Lignosulfonate (LS) and Kraft lignin (KL) with poly(methacrylic acid) (PMAA) or poly(3-sulfopropyl methacrylate), and comparing the results on concrete with respect to a commercial PCE [98]. They found that PMAA grafting significantly increased the slump of cement paste for both lignin derivatives, with LS grafted with PMAA approaching the performance of the commercial PCE superplasticizer. The researchers also measured the adsorption, zeta potential, and intrinsic viscosity of the lignopolymers to better understand the mechanisms of cement dispersion. The KL analogues adsorbed to a greater extent than the LS analogues. However, the LS analogues showed a more complex interaction with the graft chemistry, which ultimately led to the largest slump values. The researchers concluded that PMAA grafting is a promising strategy for enhancing the superplasticizer capability of lignins, but the molecular mechanisms depend strongly on the chemistry of the lignin core.

These studies suggest that lignin has the potential to be a sustainable and effective superplasticizer for concrete, but further research is needed to optimize the grafting process and to better understand the complex interactions between lignin, cement, and the grafted polymers.

All lignin modifications reported in Section 4.1 have been summarized in Table 4.

### 4.2. Economic and Environmental Perspectives of Lignin Modification Strategies

From an economic point of view, when considering the lignin modification strategies described in Section 4.1, the most commercially viable processes are sulfonation and sulfomethylation. Indeed, they rely on well-established chemical processes and highly affordable reagents, yielding water-soluble lignosulfonates at a low production cost. However, these traditional routes present severe criticisms from an environmental perspective due to the use of formaldehyde, a known human carcinogen with stringent regulations due to its belonging to the family of volatile organic compounds (VOC). This aspect inevitably compromises the “green credentials” of the resulting additives.

Regarding the oxidation processes, the use of oxygen or hydrogen peroxide obviously represents an exceptionally sustainable and low-cost approach, avoiding generally toxic organic solvents and generating minimal hazardous by-products and representing one of the most efficient, scalable, and sustainable strategies of lignin modification.

On the other hand, the exploitation of graft-copolymerization typically requires synthetic monomers, chemical initiators, and organic solvents, which increase production costs of the processes and the environmental footprint. Consequently, extensive research is currently underway in order to employ biobased monomers and sustainable reaction conditions in order to allow this strategy to be industrially and economically viable.

## 5. Quick Patent Overview

The use of lignin and its derivatives as water-reducing agents in cementitious systems has a patent history spanning nearly five decades. From a quantitative perspective, an analysis of the Google Patents database within the CPC class C04B24/18 (“lignosulfonic acid and its derivatives as organic active agents for mortars and concrete”) identifies more than 1000 documents filed between 1975 and 2024. The application field of these documents has been analyzed to extrapolate only the documents referring to LS as fluidifier/water reducer/plasticizer. This search gave a result comprising between 300 and 400 documents, which can be broadly categorized into four main technological classes: (i) unmodified lignosulfonates; (ii) chemically modified lignosulfonates; (iii) Kraft lignin-based systems, including oxidized or grafted derivatives; and (iv) composite copolymers.

Seminal patents date back to the 1970s–1980s. For instance, US4239550 [99] discloses a fluidizing agent based on mixtures of lignosulfonates with sulfonated or sulfomethylated aromatic compounds. Similarly, US4460720 [100] describes an additive for Portland cement compositions consisting of low-molecular-weight polyacrylate salts combined with lignosulfonates. Later, EP0845444 [101] introduces a formulation combining lignosulfonates with water-soluble polymers to extend workability without significantly delaying cement setting.

Patent activity remained relatively moderate until the late 1990s, when a first acceleration occurred, largely driven by efforts to mitigate the intrinsic limitations of unmodified lignosulfonates, particularly excessive air entrainment and setting retardation. In this context, US6238475 [102] reports an ammonium-based oxidative modification that significantly reduces both setting delay and air entrainment typically associated with conventional lignosulfonates.

The period 2010–2019 represents the peak of inventive activity, with a marked increase largely attributable to China. Patent trend analyses indicate that China, the United States, and Japan collectively account for more than half of all lignin-related patents. Notable Chinese contributions include: CN102936110A [103], describing a lignosulfonate–polyacrylic acid copolymer achieving water reductions of 25–40%; CN102504272B [104], which reports graft-modified lignosulfonates using acrylic acid, maleic anhydride, and polyethylene glycol (PEG), with water reduction of 22–26%; CN105271886A [105], disclosing a superplasticizer derived from oxidized alkaline lignin (MnO_2_/H_2_O_2_) with water reduction above 20%; and CN103030817A [106], focusing on modified sodium lignosulfonate as a clinker grinding aid. Additionally, CN106698994A [107] describes a graft copolymerization process between lignosulfonate and naphthalene at 85–110 °C, yielding a high-performance water reducer with improved workability and excellent slump retention.

More recent innovations are increasingly oriented toward replacing petrochemical-based admixtures with fully bio-based lignin derivatives. For example, US9676667 [108] demonstrates that unmodified Kraft or soda lignins exhibit insufficient dispersing efficiency, but that alkaline oxidation with oxygen significantly enhances their hydrophilicity and dispersant performance. In parallel, WO2015/117106 [109] shows that grafting hydrophilic polymers onto a Kraft lignin backbone via controlled radical polymerization yields water-soluble plasticizers capable of substituting conventional petrochemical surfactants in cement–water systems.

Finally, US11725130 [110] extends the application of sodium lignosulfonate as a retarding agent for oil well cementing, further confirming the technological versatility and cross-sector applicability of this class of materials.

## 6. Conclusions

The challenges of climate change caused by anthropogenic greenhouse gas emissions into the atmosphere and the growing global demand for infrastructure, driven by rapid urbanization and population growth, highlight the urgent need to introduce more sustainable technologies within the construction and civil engineering sectors (which are responsible for a considerable part of CO_2_ emissions). This impacts all aspects of the construction industry, including the development of new, sustainable concrete admixtures. In this context, technical lignins represent a potentially very interesting, environmentally friendly alternative to commercial synthetic plasticizers. However, in current commercial forms, their relatively modest performance as a concrete plasticizer is insufficient for the production of high-performance concrete, allowing their use only for conventional, low-value applications.

The works discussed in this review highlight the difficulties and, at the same time, potential routes for successfully addressing the challenge of developing lignin-based plasticizers with characteristics competitive with high-end commercial synthetic products. In particular, three fundamental ways of attacking the problem can be identified.

The first one addresses the inherent variability of the starting lignin, whose physicochemical characteristics strongly depend on the plant species of origin and the extraction process used to produce it. In this regard, several studies have demonstrated the possibility of fractionating technical lignins from different production processes with combinations of advanced techniques such as solvent extraction, membrane ultrafiltration, pH-controlled fractional precipitation, and deep eutectic solvent fractionation. These techniques have the potential to obtain lignins with characteristics tailored to specific applications and with a high degree of uniformity. This approach, however, can only lead to incremental benefits in the quality of the final cement mixture. On the other hand, the ability to develop separation processes capable of simultaneously producing different classes of molecular fractions would allow technical lignins to be valorized in various markets, increasing the value of the separation and recovery process itself.

The second approach involves improving the properties of technical lignin through sulfonation, oxidation, or carboxymethylation processes. This approach primarily allows for the conversion of widely available lignins, but unsuitable for use as a plasticizer (such as Kraft lignin), to an ordinary cement plasticizer, thus expanding the potential availability of the starting product.

The third approach, through functionalization with complex side chains, potentially allows for the generation of lignin derivatives with similar functionality to modern synthetic products (e.g., PCE) and with equivalent or superior performance. Recent research has demonstrated significant results at the laboratory level, but considerable work still remains to be done before they can be effectively scaled up to a mass industrial scale.

It’s important to note that these are not mutually exclusive strategies but represent potentially synergistic paths to achieving, in the near future, cutting-edge products that are simultaneously high-performance, sustainable, and affordable. One of the fundamental aims of this review is, indeed, to stimulate an inclusive vision of the problem that, starting from the characteristics of cement and plasticizers, considers both the upstream problems of the fractionation and refining of technical lignins and the downstream ones of the derivatization of the materials thus obtained, with a general aim of developing products and processes that are collectively sustainable from an energetic and environmental standpoint. This comprehensive approach not only allows for the exploitation of potential optimizations between the various product processing phases, but above all allows for the conception of new combined approaches that enable the design of integrated cascading processes with apparent economic and environmental advantages.

## Figures and Tables

**Table 1:** Sub-queries for the bibliometric searches in Scopus. The final search queries are constructed by combining the sub-queries with the AND operator (“ * ” is the wildcard character for Scopus searches).

Sub-Query		Scope	
		Query 1	Query 2
1	lignin OR ligno *	Title	Title
2	cement OR concrete	Title	Title/Abstract/keywords
3	water reducer OR plasticizer OR dispersant OR admixture OR surfactant	Title/Abstract/keywords	Title/Abstract/keywords

**Table 2:** Main abbreviations typically found in chemical formulae reported in works on cement chemistry.

Abbreviation	Formula	Example
H	H_2_O	CH → Ca(OH)_2_
C	CaO	C_3_A → 3CaO·Al_2_O_3_
S	SiO_2_	C_3_S → 3CaO·SiO_2_
A	Al_2_O_3_	C_2_AS → 2CaO·Al_2_O_3_·SiO_2_
F	Fe_2_O_3_	C_2_F → 2CaO·Fe_2_O_3_

**Table 3:** Comparative evaluation of native lignins: botanical origins and relative monolignol compositions.

Lignin Type	Botanical Source	Examples	H	S	G
Softwood Lignin	Gymnosperms (Conifers)	Pine, Spruce	≤5	Traces	≥90%,
Hardwood Lignin	Angiosperms (Deciduous)	Eucalyptus, Beech, Birch	traces	50–75%	25–50%
Herbaceous/Grass Lignin	Herbaceous Plants (Poaceae)	Wheat straw, sugarcane bagasse Corn stover, Bamboo	5–35%	25–50%	25–50%

**Table 4:** Summary table comparing the different lignin modification methods, the lignin types used, and their key findings.

Modification Methods	Lignin Type/ Source	Findings/Performances	Primary Mechanism	Refs
Sulfonation/ Sulfomethylation	Esparto grass soda lignin	- 0.4% dosage (*w*/*w* lignin/cement) led to a good plasticizing effect and 7–12% water reduction while maintaining workability	Electrostatic	[85]
	Bamboo residue (AL + EHR)	- AL-LG (1.6 mmol/g sulfonic groups) outperformed EHR-LG (1.0 mmol/g sulfonic groups) and commercial products in fluidity - EHR-LG increased concrete compressive strength	Electrostatic	[7]
	Eucalyptus KL	- High sulfonation degree increased water solubility from 7.2% to 98.8% - Significantly increased concrete workability	Electrostatic	[53]
	Softwood KL	- 0.5 wt% dosage increased fluidity - Performance driven by high anionic charge density (2.05 meq/g)	Electrostatic	[84]
Phenolation and sulfonation	Eucalyptus KL	- Synthesis of water-soluble SHP-SAL which showed dispersant performance comparable to synthetic SNF and better than commercial lignosulfonates	Electrostatic	[87]
Oxidation (Alkali-O_2_)	Wheat straw SL	- At 0.4 wt% dosage comparable plasticizing effect to synthetic superplasticizers	Electrostatic	[88]
Oxidation (KOH-mediated)	Softwood KL	- 18% water reduction at 0.35 wt% dosage - Compressive strength improved by 45% compared to blank at 0.25 wt% dosage	Electrostatic	[90]
Carboxymethylation	Hardwood KL	- Hardwood-derived anionic product (1.8 meq/g charge density) - Improved particle dispersion - Proposed for cement and mining industries	Electrostatic	[45,93]
	Sugarcane bagasse lignin	- 0.3 wt% dosage increased the workability of cement paste - Feasible candidate for sustainable, bio-based plasticizers	Electrostatic	[9]
Amination	Technical lignin	- Improved grindability - Improved particle size distribution (3–32 µm) - Strong retarding effect on cement hydration and setting times	Synergistic (Electro-Steric)	[94]
Graft-copolymerization	KL + TPEG	- PCE-Ls at 30% lignin content showed greater dispersion in concrete slums than lignin-free polycarboxylate references	Steric hindrance	[95,96]
	KL + LS	- PMAA grafting significantly increased cement paste slump - LS grafted with PMAA approached the performance of commercial PCE superplasticizers	Steric hindrance	[98]
	Technical Lignin	- Synthesis of lignin-based polycarboxylates provided favorable dispersion and viscosity-reducing effects	Synergistic (Electro-Steric)	[97]
DES Fractionation	Rice Husks	- Underivatized lignins from raw and parboiled husks showed water reduction performance equivalent to commercial soda lignin	Electrostatic	[43]
	Brewers’ Spent Grain	- Demonstrated water reduction capability equivalent to industrial soda lignin	Electrostatic	[44]

## Data Availability

No new data were created or analyzed in this study. Data sharing is not applicable.

## Abbreviations

The following abbreviations are used in this manuscript:

ACL	Lignin acrylate
AL	Alkaline lignin
CPC	Cooperative Patent Classification
DES	Deep Eutectic Solvents
DML	Diethanolamine-modified lignin
EHR	Enzymatic hydrolysis residue
IL	Ionic liquid
KL	Kraft lignin
KL-TPEG	Isopentenol polyoxyethylene ether-modified Kraft lignin
LS	Lignosulfonate
PCE	Polycarboxylate ether
PCE-Ls	Polycarboxylate ether-modified lignin
PMAA	poly(methacrylic acid)
SAL	Sulphuric acid lignin
SHP-SAL	Sulfonated hydroxymethylated phenolized sulphuric acid lignin
SNF	Sulfonated naphthalene formaldehyde condensate
TPEG	Isopentenol polyoxyethylene ether
*w*/*c*	Water/cement (e.g., *w*/*c* ratio)
XPS	X-ray photoelectron spectroscopy

## References

[1] Poudyal L., Adhikari K. (2021). Environmental sustainability in cement industry: An integrated approach for green and economical cement production. Resour. Environ. Sustain..

[2] Tkachenko N., Tang K., McCarten M., Reece S., Kampmann D., Hickey C., Bayaraa M., Foster P., Layman C., Rossi C. (2023). Global database of cement production assets and upstream suppliers. Sci. Data.

[3] Chen H., Li N., Unluer C., Chen P., Zhang Z.H. (2025). 200 years of Portland cement: Technological advancements and sustainability challenges. J. Clean. Prod..

[4] Prévot M.S., Finelli V., Carrier X., Deplano G., Cavallo M., Quadrelli E.A., Michel J., Pietraru M.H., Camp C., Forghieri G. (2024). An anthropocene-framed transdisciplinary dialog at the chemistry-energy nexus. Chem. Sci..

[5] Guo Y.Y., Luo L., Liu T.T., Hao L.W., Li Y.M., Liu P.F., Zhu T.Y. (2024). A review of low-carbon technologies and projects for the global cement industry. J. Environ. Sci..

[6] Friedlingstein P., O’Sullivan M., Jones M.W., Andrew R.M., Hauck J., Landschützer P., Le Quéré C., Li H.M., Luijkx I.T., Olsen A. (2025). Global Carbon Budget 2024. Earth Syst. Sci. Data.

[7] Huang Z., Wang J.Y., Bing L.F., Qiu Y.J., Guo R., Yu Y., Ma M.J., Niu L., Tong D., Andrew R. (2023). Global carbon uptake of cement carbonation accounts 1930–2021. Earth Syst. Sci. Data.

[8] Marandi N., Shirzad S. (2025). Sustainable cement and concrete technologies: A review of materials and processes for carbon reduction. Innov. Infrastruct. Solut..

[9] Akond A.U.R., Lynam J.G. (2020). Deep eutectic solvent extracted lignin from waste biomass: Effects as a plasticizer in cement paste. Case Stud. Constr. Mater..

[10] Sipponen M.H. (2026). Leveraging the molecular complexity of lignin for high-performance bio-based materials. Nat. Rev. Mater..

[11] Georgs V., Piili H., Gustafsson J., Xu C. (2025). A critical review on lignin structure, chemistry, and modification towards utilisation in additive manufacturing of lignin-based composites. Ind. Crops Prod..

[12] Kim S., Chung H.Y. (2024). Biodegradable polymers: From synthesis methods to applications of lignin-polyester. Green. Chem..

[13] Wang Y.-Y., Meng X., Pu Y., Ragauskas A.J. (2020). Recent Advances in the Application of Functionalized Lignin in Value-Added Polymeric Materials. Polymers.

[14] Kai D., Tan M.J., Chee P.L., Chua Y.K., Yap Y.L., Loh X.J. (2016). Towards lignin-based functional materials in a sustainable world. Green. Chem..

[15] Shorey R., Salaghi A., Fatehi P., Mekonnen T.H. (2024). Valorization of lignin for advanced material applications: A review. RSC Sustain..

[16] Wu Y.C., Wang H.M., Yuan L.L., Zhang Q.Q., Liu Y.Q., Shao C.Y., Hou Q.X., Sun R.C. (2024). Lignin-Based Functional Hydrogels: An Eco-friendly Bulk Material. ACS Sustain. Chem. Eng..

[17] Agustiany E.A., Rasyidur Ridho M., Rahmi D.N.M., Madyaratri E.W., Falah F., Lubis M.A.R., Solihat N.N., Syamani F.A., Karungamye P., Sohail A. (2022). Recent developments in lignin modification and its application in lignin-based green composites: A review. Polym. Compos..

[18] Yuan L.L., Wang H.M., Wu Y.C., Hou Q.X., Sun R.C. (2024). The booming lignin-derived functional composites/nanocomposites. Compos. Part B Eng..

[19] Moradi Boldaji S., Iannucci L., Grassini S., Pariani L.C., Rossato L.A.M., D’Arrigo P., Noè C., Messori M. (2026). Effect of lignin incorporation on the performance of epoxidized soybean oil biobased coatings for corrosion protection. Prog. Org. Coat..

[20] Smit A.T., Bellinetto E., Dezaire T., Boumezgane O., Riddell L.A., Turri S., Hoek M., Bruijnincx P.C.A., Griffini G. (2023). Tuning the Properties of Biobased PU Coatings via Selective Lignin Fractionation and Partial Depolymerization. ACS Sustain. Chem. Eng..

[21] Monaci S., Minudri D., Guazzelli L., Mezzetta A., Mecerreyes D., Forsyth M., Somers A. (2023). Lignin-Derivative Ionic Liquids as Corrosion Inhibitors. Molecules.

[22] Tang Q., Qian Y., Yang D., Qiu X., Qin Y., Zhou M. (2020). Lignin-Based Nanoparticles: A Review on Their Preparations and Applications. Polymers.

[23] Iravani S., Varma R.S. (2020). Greener synthesis of lignin nanoparticles and their applications. Green. Chem..

[24] Rossato L.A.M., Morsali M., Ruffini E., Bertuzzi P., Serra S., D’Arrigo P., Sipponen M. (2024). Phospholipase D Immobilization on Lignin Nanoparticles for Enzymatic Transformation of Phospholipids. ChemSusChem.

[25] Ali S., Rani A., Dar M.A., Qaisrani M.M., Noman M., Yoganathan K., Asad M., Berhanu A., Barwant M., Zhu D. (2024). Recent Advances in Characterization and Valorization of Lignin and Its Value-Added Products: Challenges and Future Perspectives. Biomass.

[26] Libretti C., Santos Correa L., Meier M.A.R. (2024). From waste to resource: Advancements in sustainable lignin modification. Green. Chem..

[27] Breilly D., Fadlallah S., Froidevaux V., Colas A., Allais F. (2021). Origin and industrial applications of lignosulfonates with a focus on their use as superplasticizers in concrete. Constr. Build. Mater..

[28] Jędrzejczak P., Collins M.N., Jesionowski T., Klapiszewski Ł. (2021). The role of lignin and lignin-based materials in sustainable construction—A comprehensive review. Int. J. Biol. Macromol..

[29] Lai G., Liu X., Li S., Xu Y., Zheng Y., Guan J., Gao R., Wei Z., Wang Z., Cui S. (2023). Development of chemical admixtures for green and environmentally friendly concrete: A review. J. Clean. Prod..

[30] Carvalho V.R., Costa L.C.B., Baeta B.E.L., Peixoto R.A.F. (2023). Lignin-Based Admixtures: A Scientometric Analysis and Qualitative Discussion Applied to Cement-Based Composites. Polymers.

[31] Ludwig H.M., Zhang W.S. (2015). Research review of cement clinker chemistry. Cem. Concr. Res..

[32] Hewlett P.C. (2019). Lea’s Chemistry of Cement and Concrete.

[33] Kurdowski W. (2014). Cement and Concrete Chemistry.

[34] Lowke D., Gehlen C. (2017). The zeta potential of cement and additions in cementitious suspensions with high solid fraction. Cem. Concr. Res..

[35] Aïtcin P.-C., Flatt R.J. (2016). Science and Technology of Concrete Admixtures.

[36] Boerjan W., Ralph J., Baucher M. (2003). Lignin biosynthesis. Annu. Rev. Plant Biol..

[37] Liu Q.Q., Luo L., Zheng L.Q. (2018). Lignins: Biosynthesis and Biological Functions in Plants. Int. J. Mol. Sci..

[38] Wang Z., Deuss P.J. (2023). The isolation of lignin with native-like structure. Biotechnol. Adv..

[39] Li T., Takkellapati S. (2018). The current and emerging sources of technical lignins and their applications. Biofuels Bioprod. Bioref..

[40] Lora J., Belgacem M.N., Gandini A. (2008). Industrial commercial lignins: Sources, properties and applications. Monomers, Polymers and Composites from Renewable Resources.

[41] Al Arni S. (2018). Extraction and isolation methods for lignin separation from sugarcane bagasse: A review. Ind. Crops Prod..

[42] Martin E., Agnihotri S., Audonnet F., Record E., Dubessay P., Taherzadeh M.J., Michaud P. (2025). From Corncob By-Product to Functional Lignins: Comparative Analysis of Alkaline and Organosolv Extraction Followed by Laccase Treatment. Biomolecules.

[43] Allegretti C., Bellinetto E., D’Arrigo P., Ferro M., Griffini G., Rossato L.A.M., Ruffini E., Schiavi L., Serra S., Strini A. (2022). Fractionation of Raw and Parboiled Rice Husks with Deep Eutectic Solvents and Characterization of the Extracted Lignins towards a Circular Economy Perspective. Molecules.

[44] Allegretti C., Bellinetto E., D’Arrigo P., Griffini G., Marzorati S., Rossato L.A.M., Ruffini E., Schiavi L., Serra S., Strini A. (2022). Towards a Complete Exploitation of Brewers’ Spent Grain from a Circular Economy Perspective. Fermentation.

[45] Dessbesell L., Paleologou M., Leitch M., Pulkki R., Xu C. (2020). Global lignin supply overview and kraft lignin potential as an alternative for petroleum-based polymers. Renew. Sust. Energ. Rev..

[46] Bajwa D.S., Pourhashem G., Ullah A.H., Bajwa S.G. (2019). A concise review of current lignin production, applications, products and their environmental impact. Ind. Crops Prod..

[47] Himmel M.E., Ding S.Y., Johnson D.K., Adney W.S., Nimlos M.R., Brady J.W., Foust T.D. (2007). Biomass recalcitrance: Engineering plants and enzymes for biofuels production. Science.

[48] Ekeberg D., Gretland K.S., Gustafsson J., Bråten S.M., Fredheim G.E. (2006). Characterisation of lignosulphonates and kraft lignin by hydrophobic interaction chromatography. Anal. Chim. Acta.

[49] Calvo-Flores F.G., Dobado J.A., Isac-García J., Martín-Martínez F.J. (2015). Lignin and Lignans as Renewable Raw Materials.

[50] Eraghi Kazzaz A., Fatehi P. (2020). Technical lignin and its potential modification routes: A mini-review. Ind. Crops Prod..

[51] Segers B., Nimmegeers P., Spiller M., Tofani G., Jasiukaityte-Grojzdek E., Dace E., Kikas T., Marchetti J.M., Rajic M., Yildiz G. (2024). Lignocellulosic biomass valorisation: A review of feedstocks, processes and potential value chains and their implications for the decision-making process. RSC Sustain..

[52] Tyagi U., Sarma A.K. (2022). Perspectives of biomass based lignin to value added chemicals in biorefineries: Challenges, extraction strategies and applications. Biofuels Bioprod. Bioref..

[53] Zandonadi Nunes C.C., de Paula H.B., Demuner I.F., de Paula M.O., Pedroti L.G., Carvalho A.M.M.L. (2024). Kraft lignin biorefinery: From pulping side streams to concrete plasticizers. Eur. J. Wood Wood Prod..

[54] Gellerstedt G. (2015). Softwood kraft lignin: Raw material for the future. Ind. Crops Prod..

[55] Aro T., Fatehi P. (2017). Production and Application of Lignosulfonates and Sulfonated Lignin. ChemSusChem.

[56] Allegretti C., Boumezgane O., Rossato L., Strini A., Troquet J., Turri S., Griffini G., D’Arrigo P. (2020). Tuning Lignin Characteristics by Fractionation: A Versatile Approach Based on Solvent Extraction and Membrane-Assisted Ultrafiltration. Molecules.

[57] Tofani G., Jasiukaityte-Grojzdek E., Grilc M., Likozar B. (2024). Organosolv biorefinery: Resource-based process optimisation, pilot technology scale-up and economics. Green. Chem..

[58] de la Torre M.J., Moral A., Hernández M.D., Cabeza E., Tijero A. (2013). Organosolv lignin for biofuel. Ind. Crops Prod..

[59] Ikeda T., Magara K. (2015). Chemical Properties of Softwood Soda-Anthraquinone Lignin. J. Wood Chem. Technol..

[60] Mousavioun P., Doherty W.O.S. (2010). Chemical and thermal properties of fractionated bagasse soda lignin. Ind. Crops Prod..

[61] Jasiukaitytė-Grojzdek E., Ročnik Kozmelj T., Tofani G., Segers B., Nimmegeers P., Billen P., Pogorevc R., Likozar B., Grilc M. (2025). Design of Organosolv Lignin Fractionation: Influence of Temperature, Antisolvent, and Source on Molecular Weight, Structure, and Functionality of Lignin Fragments. ACS Sustain. Chem. Eng..

[62] Pang T., Wang G., Sun H., Sui W., Si C. (2021). Lignin fractionation: Effective strategy to reduce molecule weight dependent heterogeneity for upgraded lignin valorization. Ind. Crops Prod..

[63] Sadeghifar H., Ragauskas A. (2020). Perspective on Technical Lignin Fractionation. ACS Sustain. Chem. Eng..

[64] Lourençon T.V., Hansel F.A., da Silva T.A., Ramos L.P., de Muniz G.I.B., Magalhaes W.L.E. (2015). Hardwood and softwood kraft lignins fractionation by simple sequential acid precipitation. Sep. Purif. Technol..

[65] Mussatto S.I., Fernandes M., Roberto I.C. (2007). Lignin recovery from brewer’s spent grain black liquor. Carbohydr. Polym..

[66] Passoni V., Scarica C., Levi M., Turri S., Griffini G. (2016). Fractionation of Industrial Softwood Kraft Lignin: Solvent Selection as a Tool for Tailored Material Properties. ACS Sustain. Chem. Eng..

[67] Allegretti C., Fontanay S., Krauke Y., Luebbert M., Strini A., Troquet J., Turri S., Griffini G., D’Arrigo P. (2018). Fractionation of Soda Pulp Lignin in Aqueous Solvent through Membrane-Assisted Ultrafiltration. ACS Sustain. Chem. Eng..

[68] Mendes S.F., Rodrigues J.S., de Lima V.H., Botaro V.R., Cardoso V.L., Reis M.H.M. (2021). Forward Black Liquor Acid Precipitation: Lignin Fractionation by Ultrafiltration. Appl. Biochem. Biotech..

[69] Yan J., Tan E.C.D., Katahira R., Pray T.R., Sun N. (2022). Fractionation of Lignin Streams Using Tangential Flow Filtration. Ind. Eng. Chem. Res..

[70] Padilha C.E.D., Nogueira C.D., Oliveira M.A., de Sousa F.C., de Assis C.F., Souza D.F.D., de Oliveira J.A., dos Santos E.S. (2019). Fractionation of green coconut fiber using sequential hydrothermal/alkaline pretreatments and Amberlite XAD-7HP resin. J. Environ. Chem. Eng..

[71] Gomes E.D., Mota M.I., Rodrigues A.E. (2018). Fractionation of acids, ketones and aldehydes from alkaline lignin oxidation solution with SP700 resin. Sep. Purif. Technol..

[72] Mqoni N., Bahadur I., Singh S., Meng X.Z., Ragauskas A. (2026). Deep Eutectic Solvents for Pretreatment of Lignocellulose Biomass: Physical Properties, Solubility Mechanisms, and Their Interactions. Chem. Rev..

[73] Amini E., Valls C., Roncero M.B. (2021). Ionic liquid-assisted bioconversion of lignocellulosic biomass for the development of value-added products. J. Clean. Prod..

[74] Halder P., Kundu S., Patel S., Setiawan A., Atkin R., Parthasarthy R., Paz-Ferreiro J., Surapaneni A., Shah K. (2019). Progress on the pre-treatment of lignocellulosic biomass employing ionic liquids. Renew. Sust. Energ. Rev..

[75] Liu Z., Hou Y., Hu S.Q. (2024). Rapid microwave-assisted pretreatment using a ternary deep eutectic solvent for lignin fractionation of eucalyptus. Ind. Crops Prod..

[76] Pariani L.C., Castiglione F., Griffini G., Rossato L.A.M., Ruffini E., Strini A., Tessaro D., Turri S., Serra S., D’Arrigo P. (2025). Synergistic DES-Microwave Fractionation of Agri-Food Biomasses in a Zero-Waste Perspective. Molecules.

[77] Chen M., Malaret F., Firth A.E.J., Verdía P., Abouelela A.R., Chen Y.Y., Hallett J.P. (2020). Design of a combined ionosolv-organosolv biomass fractionation process for biofuel production and high value-added lignin valorisation. Green. Chem..

[78] Alriols M.G., García A., Llano-ponte R., Labidi J. (2010). Combined organosolv and ultrafiltration lignocellulosic biorefinery process. Chem. Eng. J..

[79] Husanu E., Mero A., Rivera J.G., Mezzetta A., Ruiz J.C., D’Andrea F., Pomelli C.S., Guazzelli L. (2020). Exploiting Deep Eutectic Solvents and Ionic Liquids for the Valorization of Chestnut Shell Waste. ACS Sustain. Chem. Eng..

[80] Mero A., Mezzetta A., De Leo M., Braca A., Guazzelli L. (2024). Sustainable valorization of cherry (*Prunus avium* L.) pomace waste via the combined use of (NA)DESs and bio-ILs. Green. Chem..

[81] Allegretti C., Fontanay S., Rischka K., Strini A., Troquet J., Turri S., Griffini G., D’Arrigo P. (2019). Two-Step Fractionation of a Model Technical Lignin by Combined Organic Solvent Extraction and Membrane Ultrafiltration. ACS Omega.

[82] Rodrigues J.S., Lima V., Araújo L.C.P., Botaro V.R. (2021). Lignin Fractionation Methods: Can Lignin Fractions Be Separated in a True Industrial Process?. Ind. Eng. Chem. Res..

[83] Roussel N. (2012). Understanding the Rheology of Concrete.

[84] He W., Fatehi P. (2015). Preparation of sulfomethylated softwood kraft lignin as a dispersant for cement admixture. RSC Adv..

[85] Kamoun A., Jelidi A., Chaabouni M. (2003). Evaluation of the performance of sulfonated esparto grass lignin as a plasticizer–water reducer for cement. Cem. Concr. Res..

[86] Huang C., Ma J., Zhang W., Huang G., Yong Q. (2018). Preparation of Lignosulfonates from Biorefinery Lignins by Sulfomethylation and Their Application as a Water Reducer for Concrete. Polymers.

[87] Syahbirin G., Darwis A.A., Suryani A., Syafii W. (2012). Potential of Lignosulphonate of Eucalyptus Lignin from Pulp Plant as Dispersant in Gypsum Paste. Procedia Chem..

[88] Kalliola A., Vehmas T., Liitiä T., Tamminen T. (2015). Alkali-O_2_ oxidized lignin—A bio-based concrete plasticizer. Ind. Crops Prod..

[89] Kalliola A., Kangas P., Winberg I., Vehmas T., Kyllönen H., Heikkinen J., Poukka O., Kemppainen K., Sjögård P., Pehu-Lehtonen L. (2022). Oxidation process concept to produce lignin dispersants at a kraft pulp mill. Nord. Pulp Pap. Res. J..

[90] Sutradhar S., Gao W.J., Fatehi P. (2023). A Green Cement Plasticizer from Softwood Kraft Lignin. Ind. Eng. Chem. Res..

[91] Andriani F., Karlsson M., Elder T., Lawoko M. (2024). Lignin Carboxymethylation: Probing Fundamental Insights into Structure–Reactivity Relationships. ACS Sustain. Chem. Eng..

[92] Li S., Ogunkoya D., Fang T., Willoughby J., Rojas O.J. (2016). Carboxymethylated lignins with low surface tension toward low viscosity and highly stable emulsions of crude bitumen and refined oils. J. Colloid. Interface Sci..

[93] Konduri M.K., Kong F., Fatehi P. (2015). Production of carboxymethylated lignin and its application as a dispersant. Eur. Polym. J..

[94] Li Y.W., Zhu H.Y., Yang C.L., Zhang Y.F., Xu J., Lu M.G. (2014). Synthesis and super retarding performance in cement production of diethanolamine modified lignin surfactant. Constr. Build. Mater..

[95] Zheng T., Zheng D., Qiu X., Yang D., Fan L., Zheng J. (2019). A novel branched claw-shape lignin-based polycarboxylate superplasticizer: Preparation, performance and mechanism. Cem. Concr. Res..

[96] Zheng T., Zhong D., Zheng D., Bao F., Zheng J., Qiu X. (2020). Kraft lignin grafted with isopentenol polyoxyethylene ether and the dispersion performance. Int. J. Biol. Macromol..

[97] Zhong D., Liu Q., Zheng D. (2022). Synthesis of lignin-grafted polycarboxylate superplasticizer and the dispersion performance in the cement paste. Colloids Surf. A Physicochem. Eng. Asp..

[98] Childs C.M., Perkins K.M., Menon A., Washburn N.R. (2019). Interplay of Anionic Functionality in Polymer-Grafted Lignin Superplasticizers for Portland Cement. Ind. Eng. Chem. Res..

[99] Kohler L. (1980). Flowing Agent for Concrete and Mortar and Process for Producing the Same. U.S. Patent.

[100] Gaidis J.M., Rosenberg A.M. (1984). Multicomponent Concrete Superplasticizer. U.S. Patent.

[101] Bürge T.A., Mäder U., Schober I. (2002). Improved Ligninsulfonate Concrete Admixtures. European Patent.

[102] Gargulak J., Bushar L., Sengupta A. (2001). Ammoxidized Lignosulfonate Cement Dispersant. U.S. Patent.

[103] Guanghai Y., Xianghai S., Baoguo H., Hong P. (2014). Lignosulfonate-Polycarboxylic Acid Copolymerized Composite High-Performance Water Reducer and Preparation Method Thereof. China Patent.

[104] Mingshuang X., Hongyi Z., Huayu Z., Nanjing S., Fanjun M., Zongyan X. (2013). Modified Lignosulfonate Water Reducing Agent and Preparation Method Thereof. China Patent.

[105] Mingchao D., Jun Z., Yinhang J. (2015). Modified Lignosulfonate Superplasticizer and Preparation Method Thereof. China Patent.

[106] Hongman Z., He H., Fazhan Z., Jian D. (2013). Modified Sodium Lignin Sulfonate and Application Thereof as Cement Grinding Aid. China Patent.

[107] Haozhen G., Jingyuan Y. (2016). Preparation Method of Lignosulfonate Grafted Copolymerized Naphthalene-Based Concrete Water Reducer. China Patent.

[108] Kalliola A., Tiina L., Tarja T., Tapio V. (2017). Use of Oxidized Lignin as Dispersant. U.S. Patent.

[109] Washburn N.R., Gupta C. (2015). Polymer-Grafted Lignin Surfactants. International Patent.

[110] Alkhalaf S.A., Al-Yami A., Wagle V., Alsafran A. (2023). Sodium Lignosulfonate as a Retarder Additive for Oil and Gas Wells Cementing. U.S. Patent.

